# Comprehensive Analysis of Differentially Expressed Long Noncoding RNA-mRNA in the Adenoma-Carcinoma Sequence of DNA Mismatch Repair Proficient Colon Cancer

**DOI:** 10.1155/2021/9977695

**Published:** 2021-06-01

**Authors:** Wenkun Li, Qian Li, Jiang Ge, Yun Wang, Nanshan Li, Yueqiong Lao, Yadan Wang, Kuiliang Liu, Chunmei Guo, Wu Lin, Guojun Jiang, Nan Wei, Canghai Wang, Hong Liu, Jing Wu

**Affiliations:** ^1^Department of Gastroenterology, Beijing Shijitan Hospital, Capital Medical University, Beijing 100038, China; ^2^Department of Gastroenterology, Peking University Ninth School of Clinical Medicine, Beijing 100038, China; ^3^Department of Gastroenterology, Beijing Friendship Hospital, Capital Medical University, National Clinical Research Center for Digestive Diseases, Beijing, 100050, China

## Abstract

DNA proficient mismatch repair colon cancer (pMMR CC) is the most common subtype of sporadic CC. We aimed to investigate the role of long noncoding RNAs (lncRNAs) in pMMR CC carcinogenesis. In the present study, we conducted transcriptomic analysis of lncRNAs-mRNAs in five low-grade intraepithelial neoplasia (LGIN), five high-grade intraepithelial neoplasia (HGIN), four pMMR CC, and five normal control (NC) tissues. Gene Ontology (GO), Kyoto Encyclopedia of Genes and Genomes (KEGG) enrichment pathway, and coexpression network analyses were performed to elucidate the functions of lncRNAs and mRNAs as well as their interactions. Quantitative real-time polymerase chain reaction (qRT-PCR) was used to validate five dysregulated lncRNAs in a large set of colon tissues. Receiver-operating characteristic (ROC) curves were employed to evaluate the performance of the candidate lncRNAs. A set of 5783 differentially expressed lncRNAs and 4483 differentially expressed mRNAs were detected among the LGIN, HGIN, pMMR CC, and NC samples. These differentially expressed lncRNAs and mRNAs were assigned to 275 significant GO terms and 179 significant KEGG enriched pathways. qRT-PCR confirmed that the expression of five selected lncRNAs (ENST00000521815, ENST00000603052, ENST00000609220, NR_026543, and ENST00000545920) were consistent with the microarray data. ROC analysis showed that four lncRNAs (ENST00000521815, ENST00000603052, ENST00000609220, and NR_026543) had larger area under the ROC curve (AUC) values compared to serum carcinoembryonic antigens, thereby distinguishing NC from pMMR CC. In conclusion, several lncRNAs play various roles in the adenoma-carcinoma sequence and may serve as potential biomarkers for the early diagnosis of pMMR CC.

## 1. Introduction

Colorectal cancer (CRC) is the third most commonly diagnosed cancer worldwide, comprising over 1.8 million new cases in 2018 [1]. More than 70% of CRC cases are located in the colon, which is also called colon cancer (CC) [2]. According to the latest data, CRC ranks fifth in both incidence and mortality among cancers in China [3].

CRC is a heterogeneous disease with a progressive accumulation of genetic and epigenetic alterations [4–6]. About two-thirds of CRC cases arise with unknown contributions from germline factors or a significant family history of cancer or inflammatory bowel disease, defined as sporadic CRC. Three major genetic mechanisms underlie the pathogenesis of sporadic CRC [7], namely, chromosomal instability (CIN), microsatellite instability (MSI), and the epigenomic CpG island methylator phenotype (CIMP). CIN, the first described and the most common pathway, accounts for 80%–85% of sporadic CRC [8]. CIN CRCs are nonhypermutated with DNA proficient mismatch repair (pMMR) functions [7]. The mechanism responsible for CIN is different from that responsible for MSI and CIMP. Understanding the molecular mechanisms underlying CRC is critically important for the clinical prognosis and therapeutic response. A meta-analysis of 63 eligible studies (10126 patients with CRC) showed a worse prognosis for CRC patients with CIN/pMMR [9]. Additionally, stage II CRC patients with defective mismatch repair (dMMR) or high-level MSI (MSI-H) have better prognosis but no benefits from fluorouracil chemotherapy [10, 11].

More than 90% of colorectal tumorigenesis cases follow the adenoma-carcinoma sequence (ACS), with multiple gene mutations and abnormal activation of signaling pathways [12, 13]. Mutations in the adenomatous polyposis coli (APC) gene occur early during colorectal tumorigenesis, followed by the activation of the KRAS gene and the inactivation of the TP53 gene, which are often associated with CIN [14].

Long noncoding RNAs (lncRNAs) are a class of noncoding RNAs with more than 200 nucleotides that are not translated into proteins. LncRNAs can regulate gene expression at different levels, including epigenetically, transcriptionally, and posttranscriptionally [15]. Decreased expression of the lncRNA SATB2-AS1 promotes metastasis and influences the tumor microenvironment in CRC by regulating SATB2, thereby resulting in poor survival [16]. Overexpression of the lncRNA UCA1 contributes to the immune escape of cancer cells and protects PDL1 expression from miRNA repression in gastric carcinoma, serving as a potentially novel target for immunotherapy [17]. However, the differential expression and the role of lncRNAs in ACS for CRC remain to be elucidated.

Previous studies on the lncRNA expression profiles of CC were generally performed on all cancers in the colon together including rectal cancer, thereby neglecting the heterogeneity of molecular subtypes of CC. Although often linked together, CC and rectal cancer are different when it comes to treatment. Thus far, the differential expression profile for lncRNAs in the normal colonic mucosa-pMMR adenoma-pMMR sporadic CC sequence has not been reported. In the present study, we aimed to characterize the expression profile of lncRNAs in the malignant evolution process for pMMR sporadic CC using transcriptome microarray technology. The findings provide useful candidates for the diagnosis and treatment of CC.

## 2. Materials and Methods

### 2.1. Clinical Samples

A total of 244 sporadic colonic tissues, including 63 adenomas of low-grade intraepithelial neoplasia (LGIN), 32 adenomas of high-grade intraepithelial neoplasia (HGIN), 66 pMMR CC, eight dMMR CC, and 75 normal control (NC) samples from adjacent (≥10 cm) hyperplastic or inflammatory polyps were sampled at Beijing Shijitan Hospital (Beijing, China) from 2018 to 2019. The samples were immediately placed in RNAlater solution (Cat. No. 76104, Qiagen Co, GmBH, Germany) for 24 hours at 4°C. Freshly frozen samples were stored at −80°C for RNA extraction. None of the patients received targeted therapy, chemotherapy, radiotherapy, or intervention therapy. This study was approved by the ethics committee of Beijing Shijitan Hospital (No.: 2018-59) and written informed consent was obtained from all individual participants included in the study.

### 2.2. Screening of pMMR Samples and Total RNA Isolation

As in our previous study [18], multiplex PCR and immunohistochemistry were used to determine the MMR status. pMMR colonic tissues were defined as tissue samples with a low frequency of MSI (MSI-L) or microsatellite stability (MSS, no marker tested). Total RNA was extracted from the tissue samples using RNAiso Plus (Code No. 9109, TAKARA, Japan). RNA integrity was confirmed with a NanoDrop ND-2000 spectrophotometer (Thermo Fisher Scientific, Rochester, NY, USA) and Agilent Bioanalyzer 2100 (Agilent Technologies, Santa Clara, CA, US) and further purified with an RNeasy mini kit (Cat. # 74106, Qiagen, GmBH, Germany) and RNase-Free DNase Set (Cat. #79254, Qiagen Co, GmBH, Germany). RNA samples with integrity ≥7.0 and a 28S : 18S ratio ≥0.7 were used for further experiments.

### 2.3. Microarrays Analysis

The lncRNA and mRNA expression profiles in multistage colonic mucosa tissues (five NC, five LGIN, five HGIN, and four pMMR CC samples) were determined with the Agilent custom SBC Human (4*∗*180 K) lncRNA Microarray V6.0 (Product No. G4862A-074348; Agilent Technologies, Santa Clara, CA). Total RNA was amplified and labeled with the Low Input Quick Amp WT Labeling Kit (Cat. No. 5190-2943, Agilent Technologies, Santa Clara, CA, USA) following the manufacturer's instructions. Labeled cRNAs were purified with the RNeasy mini kit (Cat. No. 74106, Qiagen, GmBH, Germany). Each slide was hybridized with 1.65 *μ*g Cy3-labeled cRNA using a Gene Expression Hybridization Kit (Cat. No. #5188-5242, Agilent Technologies, Santa Clara, CA, US) in a hybridization oven (Cat. No. G2545 A, Agilent Technologies, Santa Clara, CA, USA) for 17 h. The slides were washed in staining dishes (Cat. No. 121, Thermo Shandon, Waltham, MA, US) with the Gene Expression Wash Buffer Kit (Cat. No. 5188-5327, Agilent Technologies, Santa Clara, CA, US) following the manufacturer's instructions and scanned with an Agilent Microarray Scanner (Cat. No. G2565CA, Agilent Technologies, Santa Clara, CA, USA) with default settings (Dye channel: Green, Scan resolution = 3 *μ*m, PMT 100%, 20 bit). The data were extracted with Feature Extraction Software 10.7 (Agilent Technologies, Santa Clara, CA, USA).

### 2.4. Data Processing and Identification of DEGs

#### 2.4.1. Series Test of Cluster (STC) Analysis

Raw data were normalized with the Quantile algorithm in GeneSpring Software 11.0 (Agilent Technologies, Santa Clara, CA, USA). Cluster analysis was performed to systematically and intuitively display the relatedness between samples. Differential genes expressions (DEGs) among the four groups were filtered with the F test in the random variance model (RVM) using *P* values < 0.05 and Benjamini-Hochberg false discovery rate (FDR) < 0.05 as the significant cutoff criteria [19]. STC analysis was employed to identify the most probable set of clusters responsible for the observed ACS series by profiling the dynamic nature of the temporal gene expression. NC, LGIN, HGIN, and pMMR CC were set as different points to identify significant trending models related to multistage colonic mucosa tissue and their associated DEGs by STC analysis. Clusters with *P* values < 0.05/26 were considered statistically significant.

#### 2.4.2. Gene Ontology and Kyoto Encyclopedia of Genes and Genomes Pathway Analysis

Gene Ontology (GO) analysis was performed to annotate DEGs in three categories: molecular functions, biological processes, and cellular components. Kyoto Encyclopedia of Genes and Genomes (KEGG) analysis was performed to identify the functional pathways and pathways for molecular interactions, reactions, and relation networks. The statistically significant threshold was set as *P* value < 0.01 and FDR <0.05.

### 2.5. Construction of Coexpression and Transcription Factor (TF) Regulatory Network

To identify coexpressed lncRNA-mRNA pairs and hub DEGS, the lncRNA-mRNA coexpression network was constructed based on the gene expression value and Pearson's correlation coefficient (PCC) between the differentially expressed lncRNAs and mRNAs. |PCC| ≥0.8 and *P* value < 0.01 were considered as statistically significantly relevant. In order to distinguish the TF regulatory network in the stepwise process encompassing CC progression, the interactions were predicted starting from a TF by searching the conserved TF binding sites within a putative promoter area 2000 bp upstream and 500 bp downstream of the transcriptional initiation site of coexpressed genes.

### 2.6. Validation by Quantitative Real-Time PCR (qRT-PCR)

Based on types of lncRNAs, aberrant expression (fold change), the changes in coexpression degrees, and the results of CPC, CNCI, PFAM, and PhyloCSF scores, 5 lncRNAs were selected to validate the results of microarray. The expression levels of the lncRNAs were examined by qRT-PCR in 225 colorectal tissue samples. qRT-PCR was performed with a QuantStudio^TM^ 6 Flex Real-Time PCR System (Thermo Fisher, Rochester, NY, USA). Primers are listed in Additional file 1 and glyceraldehyde 3-phosphate dehydrogenase (GAPDH) was used as the endogenous control. Each qRT-PCR reaction (in 10 *μ*L) contained 5 *μ*L 2X SYBR Green PCR Mix (ABI, USA), 0.4 *μ*M of each of the forward and reverse primers, and 5 ng of template cDNA. The PCR program was as follows: initial denaturing at 95°C for 5 min followed by 40 cycles at 94°C for 30 s and 60°C for 1 min and fluorescence acquisition at 60°C for 1 min. PCR amplifications were performed in triplicate for each sample.

### 2.7. Statistical Analysis

Computer-based calculations were conducted using SPSS version 20.0 (SPSS Inc., Chicago, IL, USA) and MedCalc version 19.1 (MedCalc Software Bvba, Ostend, Belgium). The differences in the expression of selected lncRNAs among multiple groups were compared using ANOVA. Further pairwise comparisons were performed with the least significance difference (LSD) test. The differences in the expression of lncRNAs between the pMMR CC group and the dMMR CC group were compared with the nonparametric Mann–Whitney *U* test. The relationship between lncRNAs and their target coding genes were determined by Spearman correlation analysis. Receiver-operating characteristic (ROC) curves and the area under the ROC curve (AUC) were employed to evaluate the diagnostic accuracy of candidate lncRNAs serum carcinoembryonic antigen (CEA), obtained from Hospital Information System. The data were presented as the mean with standard deviation (SD) for normally distributed data and the median with the interquartile range for skewed data. |Fold change| ≥5 was used as the significant threshold to screen differentially expressed lncRNA and mRNA. All *P* values were two-sided, and FDR was calculated for multiple comparisons. Differences with *P* < 0.05 were considered as statistically significant.

## 3. Results

### 3.1. Basic Characteristics of the Microarray Data

**Figure 1:**
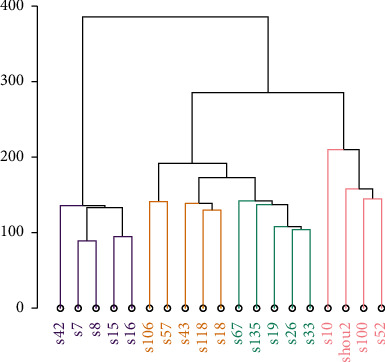
Cluster of samples. Purple represents NC group, yellow represents LGIN group, green represents HGIN group, and pink represents CC group.

The total RNA extracted from pMMR colonic tissue samples, consisting of five NC, five LGIN, five HGIN, and four pMMR CC samples, were hybridized to a transcriptome microarray. The quality control results for RNA/microarray are summarized in Additional file 1 and Additional file 2, respectively. Cluster analysis of normalized data indicated that the samples from each group were well separated when all DEGs were considered (Figure 1), suggesting that the microarray data were reliable for further bioinformatics analysis.

### 3.2. Dysregulated lncRNAs and mRNAs in the Colonic Adenoma-Carcinoma Sequence

**Figure 2:**
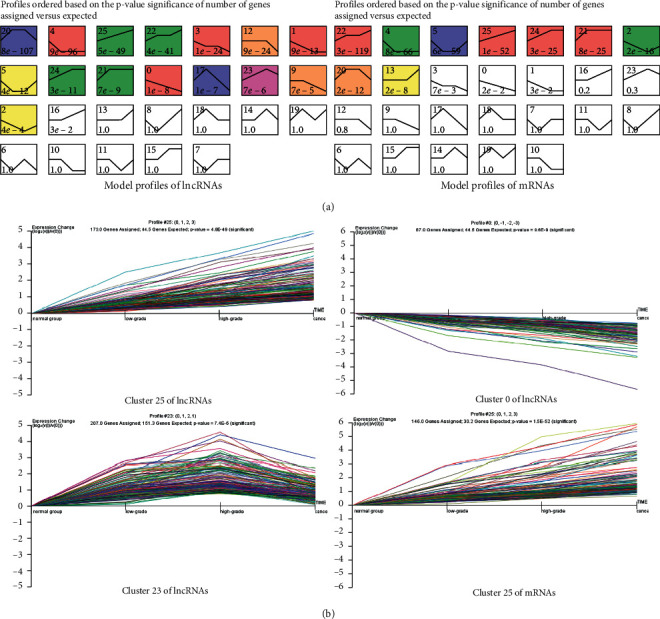
STC analysis of the dysregulated DEGs in multistage colonic mucosa tissues. (a) Twenty-six clusters for the expression pattern of DEGs, 15 lncRNA expression patterns, and nine mRNA expression patterns showed significant *P* values (colored boxes, *P* < 0.05/26). Each box represents a model expression profile, the lower number in the profile box is the *P* value, and the upper number is the model profile number. (b) Cluster 25 (173 lncRNAs) gradually increased, cluster 0 (87 lncRNAs) showed a decreasing trend, cluster 23 contained 207 lncRNAs that gradually increased from NC to adenoma and then decreased in CC, and cluster 25 (146 mRNAs) gradually increased. Horizontal axis represents different stages of colonic mucosal protruding lesions and the vertical axis shows the time series for the lncRNA/mRNA expression levels. *r* normalized with log transformation.

A total of 5783 lncRNAs and 4483 mRNAs were differentially expressed among the NC, LGIN, HGIN, and pMMR CC samples. In order to generate dynamic and significant trending models of DEGs related to colon mucosa malignant transformation, STC analysis was used to further identify the clusters of DEGs with similar expression patterns. As shown in Figure 2(a), the differential expression profile consisted of 26 differentially expressed lncRNA clusters, among which 15 clusters (profile no. 0, 1, 2, 3, 4, 5, 9, 12, 17, 20, 21, 22, 23, 24, and 25) showed significant expression trends (*P* < 0.05). Moreover, the lncRNA expression levels in clusters 21, 22, 24, and 25 were stable or gradually elevated, while the lncRNA expression in clusters 0, 1, 3, 4, 9, and 12 showed a decreasing trend. Cluster 23 contained 207 lncRNAs that gradually increased from NC to adenoma and were suppressed in CC (Figure 2(b)). However, lncRNA expression in cluster 3 was opposite that in cluster 23. As shown in Figure 2(a), a total of nine mRNA expression clusters, specifically clusters 2, 4, 5, 13, 20, 21, 22, 24, and 25, showed significant expression trends (*P* < 0.05), among which clusters 21, 22, 24, and 25 showed a stable or gradual elevation. The results suggested that adenoma is an intermediate step from normal tissue to CC, and certain lncRNAs may play important roles during the dynamic process in colonic mucosal protruding lesions.

### 3.3. Functional and Pathway Enrichment Analysis

**Figure 3:**
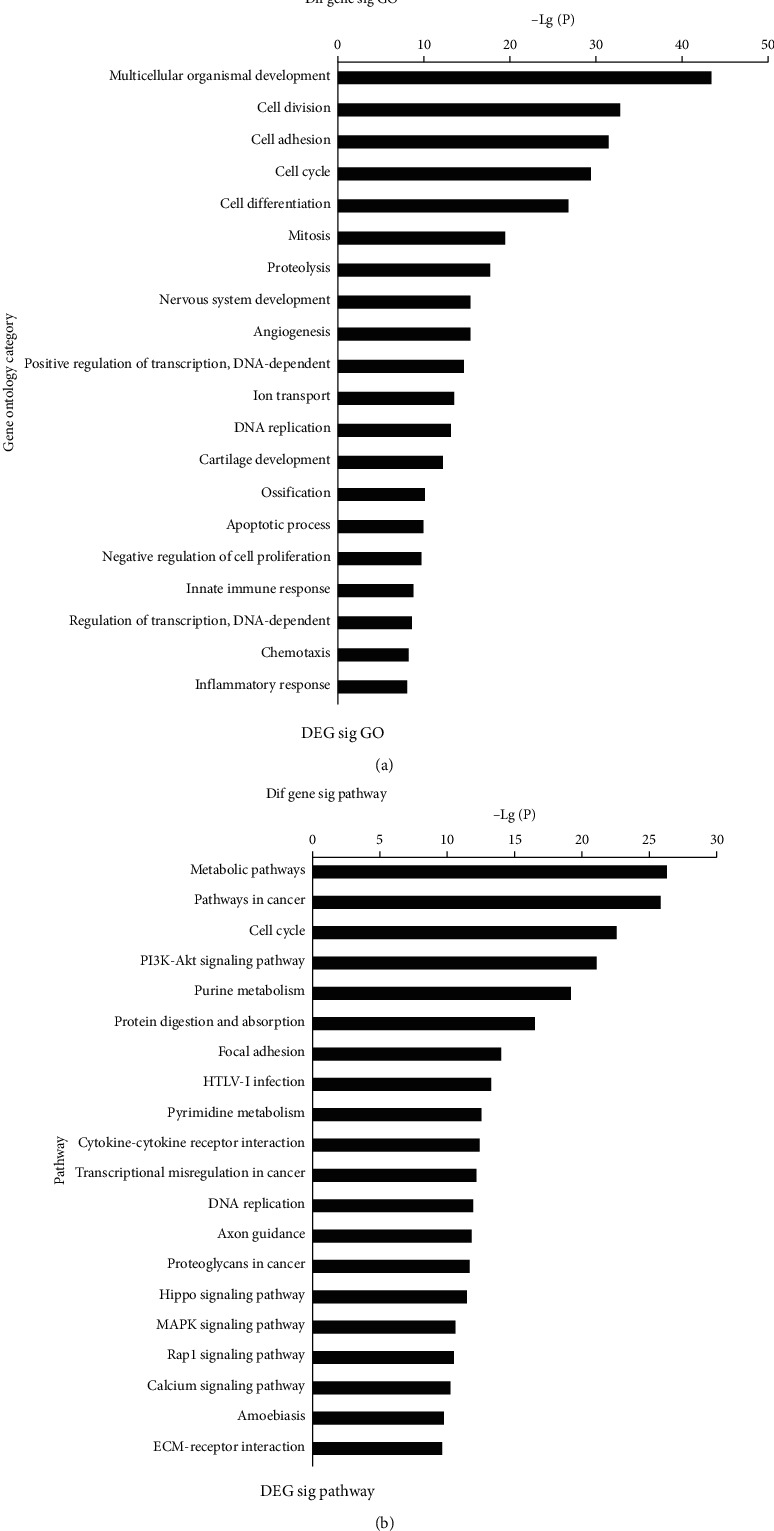
Overview of GO annotation and KEGG enrichment pathways. (a) Top 20 GO terms for DEGs. (b) Top 20 pathways corresponding to DEGs. *P* value < 0.05 and FDR <0.05.

DEGs from significant expression trends were subjected to GO and KEGG pathway analyses to identify their potential functions and mechanisms in the dynamic process for colonic mucosal protruding lesions (Figure 3).

The top 20 GO terms included multicellular organismal development, cell division, cell adhesion, cell cycle, cell differentiation, mitosis, proteolysis, and angiogenesis. KEGG enrichment analysis revealed that the DEGs were mainly involved in several signaling pathways, including metabolism, cancer, cell cycle, PI3K-Akt signaling, and transcriptional misregulation in cancer (Figure 3(b)).

### 3.4. Construction of the lncRNA-mRNA Coexpression networks

**Figure 4:**
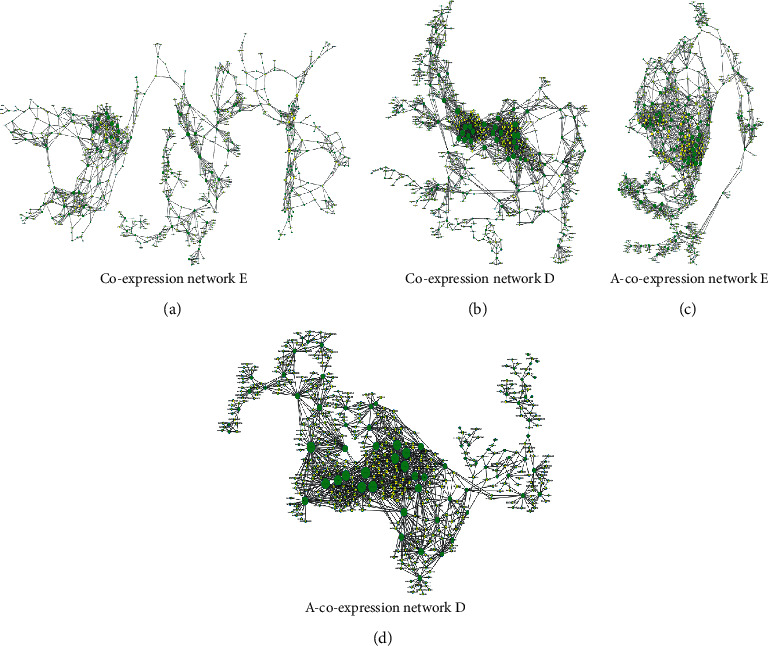
lncRNA-mRNA coexpression networks. (a-b) LncRNAs with elevated or decreased profiles and mRNAs for significant terms from GO and KEGG enrichment analysis were, respectively, selected to construct coexpression networks E and D in colonic ACS. (c-d) LncRNAs with significantly elevated/decreased model profiles and angiogenesis-related GO and KEGG items were, respectively, selected to construct A-coexpression networks E and D in colonic ACS. Nodes represent DEGs (green for mRNAs and yellow for lncRNAs). Lines represent interactions and node size represents degree value.

To discover the significant molecular mechanisms for the lncRNAs associated with tumorigenesis in colonic ACS, lncRNAs from elevated clusters [20–23] or reduced clusters (0, 1, 3, 4, 9, and 12) and mRNAs with significant GO terms and KEGG enrichment pathways were selected to construct elevated-lncRNA-mRNA coexpression networks (coexpression network E) and decreased-lncRNAs-mRNA coexpression networks (coexpression network D), respectively. The top 10 lncRNA/mRNA in terms by degree are listed in Tables 1 and 2. Coexpression network E contained 714 nodes and 1711 edges (Figure 4(a)), among which the hub nodes with the highest degrees were NOTCH4 (mRNA, degree = 29), INHBA (mRNA, degree = 26), TSTA3 (mRNA, degree = 29), lnc-NPRL3-1:1 (lncRNA, degree = 22), NR_024431 (lncRNA, degree = 21), and ENST00000564984 (lncRNA, degree = 17). Coexpression network D contained 598 nodes and 1908 edges (Figure 4(b)) and the DEGs with the highest degrees were AQP8 (mRNA, degree = 47), STRADB (mRNA, degree = 47), ENST00000435912 (lncRNA, degree = 15), NR_024431 (lncRNA, degree = 21), and ENST00000564984 (lncRNA, degree = 17) (Tables 1 and 3).

Angiogenesis is a crucial step in tumor growth and progression. The angiogenic switch has been observed at the adenoma stage in ACS [24]. We constructed angiogenesis-related lncRNA-mRNA coexpression networks (A-coexpression networks E and D) with DEGs from significantly elevated/decreased clusters as well as GO terms and KEGG pathways related to angiogenesis. A-coexpression network E consisted of 649 nodes and 2299 edges (Figure 4(c)) and the top hub genes were NOTCH4 (mRNA, degree = 35), MAD2L1 (mRNA, degree = 34), E2F7 (mRNA, degree = 34), lnc-ZBTB20-2:1 (lncRNA, degree = 22), lnc-CENPH-2:1 (lncRNA, degree = 20), and lnc-PAQR4-2:1 (lncRNA, degree = 19) (Tables 2 and 3). The top hub genes in A-coexpression network D (Figure 4(d)) were CAMK2B (mRNA, degree = 61), STRADB (mRNA, degree = 60), LTK (mRNA, degree = 57), ENST00000513255 (lncRNA, degree = 12), NR_024605 (lncRNA, degree = 11), and NONHSAT057082 (lncRNA, degree = 11) (Tables 2 and 3). All the DEGs primarily participated in angiogenesis as the key genes in each network.

### 3.5. Construction of TF Regulatory Networks

**Figure 5:**
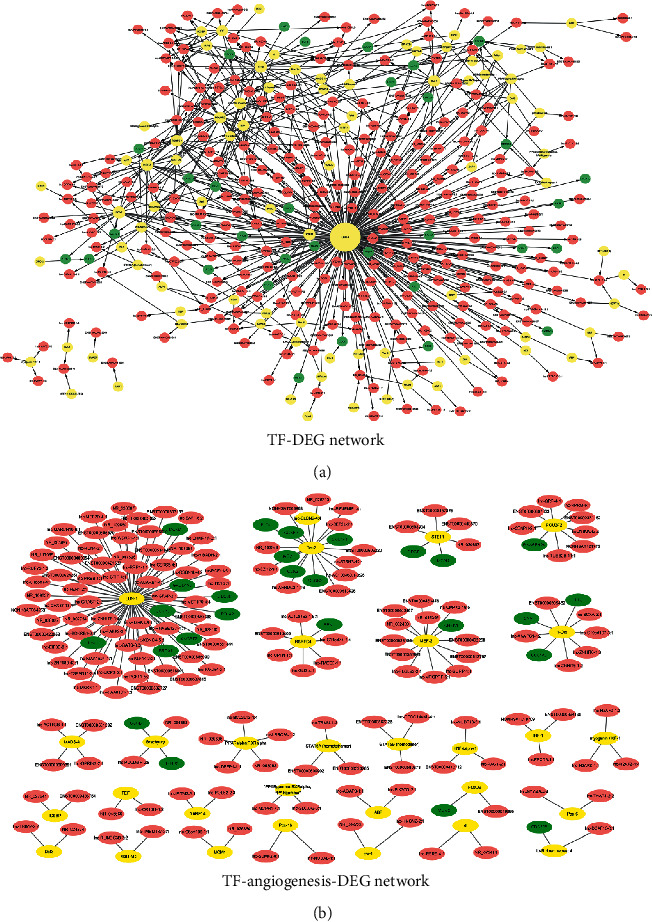
Transcription factor (TF) regulatory network. (a) Interaction networks between TFs and DEGs that originated from significant model profiles. (b) Interaction networks between TFs and DEGs that originated from angiogenesis-related model profiles. The yellow-colored nodes represent TFs, the red-colored nodes represent lncRNAs, and the green-colored nodes represent mRNAs.

To investigate the mechanism of gene regulation at the transcriptional level, we constructed two interaction networks (TF-DEG network (Figure 5(a)) and TF-angiogenesis-DEG network (Figure 5(b))) between TFs and DEGs that originated from the coexpression network or A-coexpression network. The TF-DEG network contained 99 regulation models and 402 nodes involving 85 TFs (Figure 5(a)), among which three TFs, namely, LUN-1 (TF, degree = 218), Tel-2 (TF, degree = 22), and 1-Oct (TF, degree = 20), were regulated over 20 nodes (Table 4). LUN-1 had the highest degree and might play more important roles in the TF regulatory networks. The lncRNAs regulated by the most TFs were NR_103548, lnc-ARRDC3-1:16, ENST00000513626, and ENST00000516496 (regulated by six TFs). In the TF-angiogenesis-DEG network, due to the important role of LUN-1 (TF, degree = 73) and Tel-2 (TF, degree = 18) in the progression of CC, a subnetwork was constructed showing their effects on angiogenesis functions (Figure 5(b) and Table 4).

### 3.6. qRT-PCR Verification for the Candidate Genes

**Figure 6:**
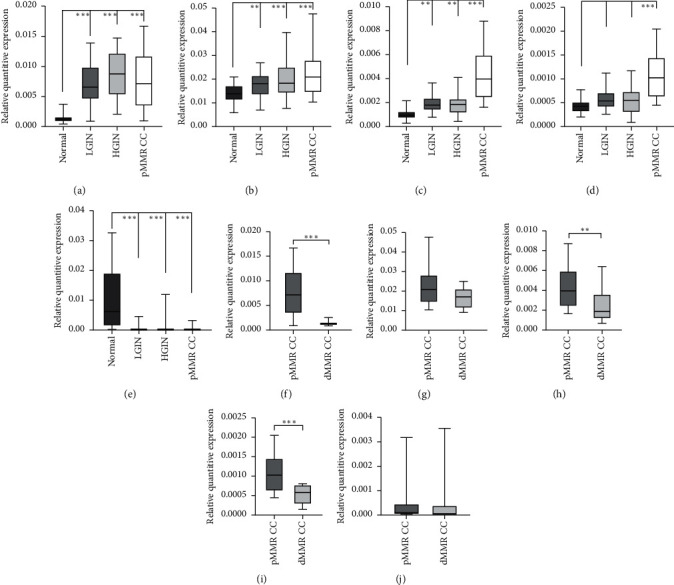
qRT-PCR verification of the candidate genes. ENST00000545920 (a), ENST00000521815 (b), ENST00000609220 (c), and ENST00000603052 (d) presented a sequentially ascending trend in expression with tumor progression, whereas NR_026543 (e) showed a sequentially decreasing trend. The expression of ENST00000521815 (f), ENST00000603052 (h), and ENST00000609220 (i) showed statistically significant differences between pMMR CC and dMMR CC. However, the expression of ENST00000609220 (g) and NR_026543 (j) did not show statistically significant differences between two groups.

According to the probe signal value, type, DEG fold change, degree value, and protein coding ability, five LncRNAs were selected from the networks above for validation in the expanded clinical samples (70 NC, 58 LIGN, 27 HIGN, 62 pMMR CC, and 8 dMMRCC samples). LncRNAs ENST00000545920, ENST00000521815, ENST00000609220, and ENST00000603052 presented a sequentially increasing trend in expression with tumor progression (Figures 6(a)–6(d)), whereas NR_026543 showed a sequentially decreasing trend (Figure 6(e)). No statistically significant differences were observed in the expressions of NR_026543 and ENST00000521815 among LGIN, HGIN, and pMMR CC. However, the expression levels for each group were significantly elevated or decreased compared with those of the NC samples, suggesting that these genes could be used to distinguish normal from lesion tissue. Furthermore, the expressions of ENST00000603052, ENST00000521815, and ENST00000609220 were significantly different in pMMR CC and dMMR CC tissue samples (Figures 6(f)–6(j)), indicating that these three genes could distinguish pMMR CC from dMMR CC.

### 3.7. Diagnostic Efficacy of Selected lncRNAs

**Figure 7:**
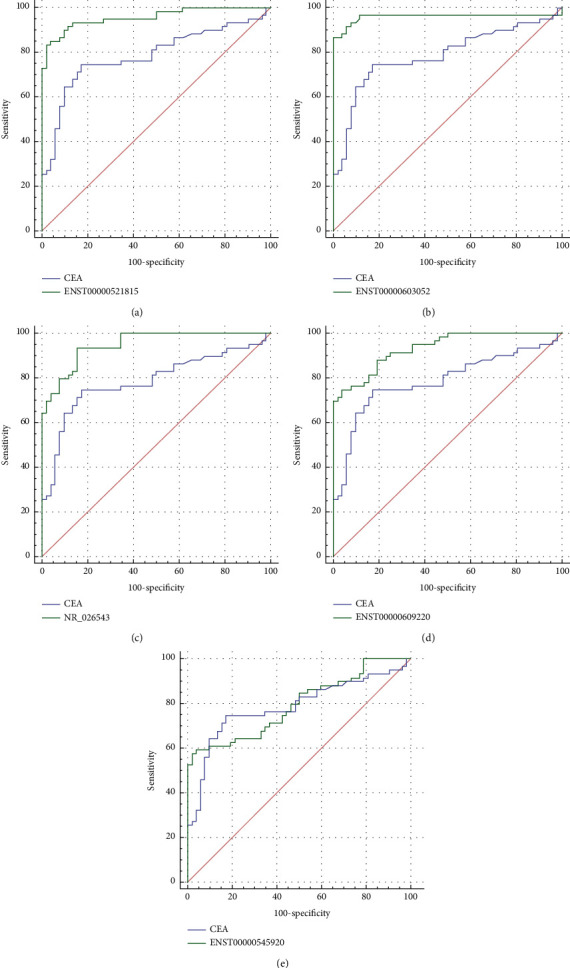
Diagnostic efficacy of selected lncRNAs. Compared with CEA, ENST00000521815 (a), ENST00000603052 (b), ENST00000609220 (c), and (d) NR_026543 had significantly larger AUCs (AUCs 0.957, 0.958, 0.933, and 0.949 versus 0.785, *P*=0.0001, 0.0002, 0.0028, and 0.0011, respectively). However, there were no statistically significant differences between the AUC of ENST00000545920 (0.794) and that of CEA (*P*=0.8734) (e). The ROC curve for CEA is shown in blue, while the ROC curves for the validation of the lncRNAs are shown in green.

The diagnostic efficacy of the above selected five lncRNAs was evaluated as potential biomarkers for pMMR CC diagnosis compared to carcinoembryonic antigen (CEA), a traditional serum tumor biomarker. The specificities of ENST00000521815, ENST00000603052, ENST00000609220, NR_026543, ENST00000545920, and CEA were 0.981, 1, 0.962, 0.932, 0.981, and 0.904, and the sensitivities were 0.931, 0.864, 0.746, 0.846, 0.576, and 0.593, respectively. ROC analysis showed significantly higher AUC for lncRNAs ENST00000521815, ENST00000603052, ENST00000609220, and NR_026543 compared with CEA (0.785) (*P*=0.0001, 0.0002, 0.0028, and 0.0011, respectively) (Figures 7(a)–7(d)). No statistically significant difference in the AUC was observed between ENST00000545920 (0.794) and CEA (*P*=0.8734) (Figure 7(e)).

## 4. Discussion

Among the human transcriptome, more than 90% are noncoding RNAs (ncRNAs), including microRNAs (miRNAs), lncRNAs, and circular RNAs (circRNAs) [20]. As a relatively new field, accumulated studies have uncovered the crucial roles of lncRNAs in oncogenicity [21, 25]. With the improvement of microarray and next-generation sequencing technology, large-scale aberrantly expressed lncRNAs have been discovered in multiple cancers, including breast cancer [22], gastric cancer [23], CRC [26], lung cancer [27], and pancreatic cancer [28].

A large proportion of colon adenomas are able to transform into adenocarcinoma through a dynamic process with the accumulation of multiple mutations and preternatural activation of signaling pathways [13]. Altered lncRNA expression patterns for microsatellite-stable colorectal cancer indicated that several lncRNAs were continuously upregulated/downregulated during the adenoma-adenocarcinoma sequence [29]. Unlike previous studies that focused on one or more lncRNAs in CRC [30–32], we first conducted a systematic and comprehensive analysis using microarrays to reveal the differentially expressed profiles of lncRNAs and mRNAs in the malignant evolutionary process for pMMR CC. Thousands of dysregulated transcripts were identified in the ACS, including 5783 lncRNAs and 4483 mRNAs. Subsequently, STC analysis revealed that adenoma is an intermediate step from normal tissue to CC and certain lncRNAs participate in this continuous process. As the dysregulation can be detected already in adenomas, these lncRNAs can be used as biomarkers for the screening and early detection of CC.

GO annotation and KEGG pathway analysis were performed to determine the functions underlying the differentially expressed lncRNAs in ACS. GO annotation suggested that aberrant transcripts were predominantly enriched in multicellular organismal development, cell division, cell adhesion, cell cycle, cell differentiation, mitosis, proteolysis, and angiogenesis. KEGG enrichment analysis revealed that the dysregulated transcripts mainly participated in metabolic pathways, pathways in cancer, cell cycle, PI3K-Akt signaling pathway, and purine metabolism. Cell cycle involvement was identified in both analyses. The uncontrolled tumor cell cycle is an essential feature of cancer and is caused by aberrant cell cycle proteins including cyclin-dependent kinases, Aurora kinases, and Polo-like kinases. Exploiting key molecules in the cell cycle may provide a new perspective for cancer therapy [33].

To further explore the underlying functions of lncRNAs in ACS, coexpression of lncRNAs with their associated coding genes was performed based on the degree of correlation. Among the top 10 elevated and decreased lncRNAs according to the degree, NR_029374 with 12 degrees was upregulated, consistent with the results obtained by Sun et al. [34] who reported that NR_029374 was upregulated in CC and correlated with poor overall survival. NR_029374 promoted the migration, invasion, and metastasis of CC through the miR-30-5p/SOX9 axis and this finding was supported by previous results [35]. NR_029374 was upregulated in hepatocellular carcinoma (HCC), markedly promoting HCC proliferation, migration, and angiogenesis [35].

Angiogenesis plays a key role in tumorigenesis and development by delivering nutrients and evacuating metabolic wastes [36]. Recently, several lncRNAs and mRNAs were demonstrated to participate in tumor angiogenesis. Upregulation of the lncRNA FLANC promoted angiogenesis by upregulating and prolonging the half-life of phosphorylated STAT3/VEGFA in CRC [37]. In the present study, we discovered that NOTCH4 (mRNA, degree = 35), a member of the Notch family of transmembrane receptors, had the highest degree. Wu et al. reported the association of overexpressed NOTCH4 with CRC survival [38]. The NOTCH4 expression level was especially higher in non-small-cell lung cancer tissues than in the NC tissues, related to the tumor size and TNM stage [39]. Active NOTCH4 might inhibit endothelial sprouting in vitro and vivo [40]. Further study is required to determine the expression level of NOTCH4 in a large population of pMMR CC patients and the underlying interactions with lncRNAs in angiogenesis. Several lncRNAs have demonstrated interactions with TFs in tumorigenesis and progression. For instance, lncRNA SNHG15 obstructed the ubiquitination and degradation of Slug, a fast-turnover TF critical for controlling cancer cell invasion and metastasis, thereby promoting CC progression [41]. The transcription of lncRNA LINC01503 was activated by TF TP63, resulting in shorter survival times for patients with esophageal squamous cell carcinoma [42]. In the current study, the lncRNA-TF network analysis revealed that LUN-1 had the highest degree in both the TF-DEG network and the TF-angiogenesis-DEG network.

qRT-PCR analysis showed the upregulation of ENST00000545920, ENST00000521815, ENST00000609220, and ENST00000603052 and the downregulation of NR_026543 with tumor progression. Furthermore, ENST00000603052, ENST00000521815, and ENST00000609220 can be used to identify pMMR CC and dMMR CC. Previous studies revealed the upregulation of ENST00000545920, also known as lnc-SNHG1, in CRC [41], non-small-cell lung cancer [43], gastric cancer [44], and glioma [45] to promote cancer cell growth by interacting with EZH2 in the nucleus and miR-154-5p in the cytoplasm [46]. Similar results have been observed in other tumors, including ENST00000521815, also known as CASC19, which possesses an oncogenic function through targeting miR-140-5p/CEMIP in CRC progression [47]. The downregulation of NR_026543 was associated with liver metastasis and poor prognosis for CC by binding with the miR-203 promoter [48]. ENST00000609220 was found to be highly expressed in CRC tissue, promoting CRC growth and metastasis through the miR-206/YAP1 axis [49]. To date, there is little knowledge on the function of ENST00000603052 in cancer.

Ultimately, the diagnostic efficacy of the identified lncRNAs was evaluated against serum CEA. Most of the selected lncRNAs achieved a slightly higher AUC in distinguishing CC from normal tissue. Further study is needed to validate these lncRNAs as biomarkers for the early diagnosis of CC by measuring their expression in serum samples to assess the consistency in tissue samples. Monitoring expression changes in these genes may also provide a new strategy to assess disease progression.

There are certain limitations in the present study. First, the sample size was relatively small, thereby limiting result reliability. We increased the number of clinical samples to confirm the function of the lncRNAs. Second, we only constructed expression profiles for colon tissue. Additional studies are required to evaluate tissues from other tumors. Last, compared with high-throughput sequencing, microarray is incapable of identifying novel lncRNAs.

## 5. Conclusions

In conclusion, we verified a series of differentially expressed lncRNAs and mRNAs in a normal mucosa-adenoma-pMMR CC sequence in the current study. The potential roles of these RNAs were predicted through bioinformatics analyses. The findings may provide novel insights for the diagnosis and therapeutic strategy of pMMR CC. Further studies are required to provide robust validation evidence for the functions of lncRNAs in CC.

## Figures and Tables

**Table 1:** The top 10 lncRNAs with the highest degree in coexpression network E/D

Gene	NC	LGIN	HGIN	CC	*P* value	FDR	Type	Degree
LncRNAs of elevated profiles								
Lnc-NPRL3-1:1	1625.59	2834.43	2901.7	2777.36	0.0037928	0.0253	Sense	22
NR_024431	7.42	18.65	19	18.6	0.0035591	0.0242	Intergenic	21
ENST00000564984	9.17	18.63	19.15	18.83	0.002729	0.0204	Antisense	17
Lnc-CLEC3A-9:1	10892.39	20059.59	19686.96	19862.6	0.0007135	0.00894	Sense	13
NR_029374	17.3	41.96	42.92	39.14	0.0064708	0.0358	Antisense	12
Lnc-MKI67IP-8:1	25.14	43.39	43.53	40.98	0.0044777	0.0282	Antisense	12
NONHSAT064233	60.28	118.49	113.3	273.65	1.68e-005	0.000976	Antisense	11
Lnc-DLX2-9:1	13.74	32.64	32.78	30.29	0.0038192	0.0254	Antisense	11
ENST00000545920	202.78	259.09	260.38	421.62	0.0019042	0.0162	Bidirectional	10
Lnc-MAP3K9-9:1	406.91	663.73	501	1392.28	0.0010763	0.0114	Antisense	10

LncRNAs of descending profiles								
ENST00000435912	35.42	19.61	19.43	19.98	0.0083754	0.0425	Antisense	15
Lnc-PDZK1-1:1	1838.67	149.47	119.91	220.16	0.0004298	0.00669	Antisense	14
Lnc-MRPL54-2:1	200.94	123.77	123.05	125.75	0.0106357	0.0495	Sense	14
ENST00000364025	7.73	3.84	3.78	3.92	0.0026283	0.0199	Sense	14
Lnc-CCNB3-2:1	617.57	101.05	95.87	115.2	4.63e-005	0.00179	Intergenic	14
Lnc-CCDC14-2:2	68.74	7.14	5.92	10.93	5.71e-005	0.00201	Sense	13
NR_003064	29637.97	1140.93	703.58	1579.91	6e-007	0.000143	Intergenic	13
Lnc-SRSF5-1:1	8.49	3.84	3.77	4.14	1.61e-005	0.000956	Intergenic	13
NR_110552	472.62	9.51	10.64	39.1	0.0001868	0.00406	Antisense	13
Lnc-ATF6B-1:4	41.99	5.78	5.88	8.03	3.64e-005	0.00154	Sense	13

**Table 2:** The top 10 mRNAs with the highest degree in coexpression networks.

Coexpression network E		Coexpression network D		A-coexpression network E		A-coexpression network D	
NOTCH4	29	AQP8	47	NOTCH4	35	CAMK2B	61
INHBA	26	STRADB	47	MAD2L1	34	STRADB	60
TSTA3	24	GRIA3	46	E2F7	34	LTK	57
RFC3	24	SCN7A	46	CCNE1	32	PDZK1	56
POLR3K	24	AGTR1	46	INHBA	32	FGF18	55
CCNE1	24	EEF2K	46	RNASEH2A	30	SEMA3E	54
ZYX	23	ALPI	46	RFC3	29	SCIN	54
ALYREF	23	CAMK2B	44	CLSPN	29	SST	53
BRCA2	22	CNTN1	44	NME1	29	IGF1	53
MAD2L1	21	IGF1	44	ERCC6L	28	BMP3	52

**Table 3:** The top 10 lncRNAs with the highest degree in A-coexpression network E/D.

Gene	NC	LGIN	HGIN	CC	*P* value	FDR	Type	Degree
LncRNAs of elevated profiles								
Lnc-ZBTB20-2:1	32.95	48.38	41.53	94.12	0.0001576	0.00367	Sense	22
Lnc-CENPH-2:1	718.1	1438.07	1182.1	3286.29	0.0003228	0.0056	Sense	20
Lnc-PAQR4-2:1	450.98	753.46	692.77	1489.34	0.0032726	0.0229	Sense	19
Lnc-SRGN-1:2	3.78	6.76	5.07	16.83	0.0059561	0.034	Intergenic	19
NR_045669	94.62	135.86	126.36	246.74	8.6e-006	0.000666	Antisense	18
Lnc-GRASP-2:2	5	13.63	12.86	40.9	0.0012449	0.0125	Sense	18
NR_036480	90.08	160.88	152.83	351.49	0.0001743	0.00391	Antisense	18
Lnc-BDKRB1-3:1	1199.21	1623.17	1474.82	2825.3	0.0005168	0.00739	Sense	18
Lnc-TXNDC3-1:1	146.23	288.55	263.33	624.69	0.001605	0.0146	Intergenic	17
ENST00000455309	140.37	210.07	197.67	369.84	0.0093281	0.0455	Antisense	17

LncRNAs of descending profiles								
ENST00000513255	193.19	58.84	55.95	67.08	<1e-07	5.34e-005	Antisense	12
NONHSAT057082	28.11	6.37	6.24	7.91	2.31e-005	0.00117	Sense	11
Lnc-SRSF5-1:1	8.49	3.84	3.77	4.14	1.61e-005	0.000956	Intergenic	11
Lnc-CCDC14-2:2	68.74	7.14	5.92	10.93	5.71e-005	0.00201	Sense	11
ENST00000617921	173.45	13.12	9.11	22.58	1.7e-006	0.00027	Intergenic	11
Lnc-RAG1-5:1	427.82	137.02	132.72	154.63	0.000499	0.00724	Sense	10
Lnc-TMTC3-5:4	84.52	4.05	4.46	8.36	<1e-07	<1e-07	Intergenic	10
Lnc-SLC10A6-5:1	32.91	12.69	12.13	13.98	0.0044594	0.0281	Sense	10
Lnc-GCNT1-6:1	24.59	4.32	4.44	5.47	<1e-07	<1e-07	Sense	10
NR_126337	101.45	35.03	33.64	38.33	0.0048936	0.0299	Intergenic	10

**Table 4:** Key transcription factors (TFs) in regulatory networks.

TFs	Degree	TFs	Degree
TF-DEGs network		TF-angiogenesis-DEGs network	
LUN-1	218	LUN-1	73
Tel-2	22	Tel-2	18
1-Oct	22	MEF-2	13
RSRFC4	20	POU3F2	9
POU3F2	20	1-Oct	9
MEF-2	20	RSRFC4	7
STE11	19	PPAR-alpha	5
Evi-1	18	MADS-A	4
STAT5B	16	Brachyury	4
IRF-1	12	STAT5A	3

## Data Availability

All the data generated or analyzed during this study are included in this published article and its supplementary information files.

## Supplementary Materials

Supplementary Materials Additional file 1. Quality control results of sample RNA. Additional file 2. Quality control results of microarrays.
