# Supplementary material for: Comprehensive Analysis of Differentially Expressed Long Noncoding RNA-mRNA in the Adenoma-Carcinoma Sequence of DNA Mismatch Repair Proficient Colon Cancer
Source: J Oncol. 2021 Jun 1;2021:9977695. doi: 10.1155/2021/9977695 (PMC8208869; doi:10.1155/2021/9977695)
Supplement: Supplementary Materials — Additional file 1. Quality control results of sample RNA. Additional file 2. Quality control results of microarrays. [file 9977695.f1.zip › 9977695.f1/Additional file 1.docx]

**Additional file 1.** **Quality control results of sample RNA**

| Group | Sample | concentration(ng/μL) | Volume(μL) | Total(μg) | A260/ A280 | 2100 Result | |
| --- | --- | --- | --- | --- | --- | --- | --- |
|  |  |  |  |  |  | RIN | 28S/18S |
|  | 7 | 255.7 | 45 | 11.51 | 1.94 | 7.4 | 1.5 |
|  | 8 | 159.9 | 100 | 15.99 | 1.91 | 7.2 | 1.8 |
|  | 15 | 202.7 | 100 | 20.27 | 1.93 | 7.0 | 1.4 |
|  | 16 | 208.1 | 45 | 9.36 | 1.94 | 7.3 | 1.7 |
| NC | 42 | 210.8 | 100 | 21.08 | 1.92 | 8.0 | 1.3 |
|  | 67 | 368.6 | 300 | 110.58 | 2.00 | 8.2 | 1.3 |
|  | 135 | 251.3 | 100 | 25.13 | 1.93 | 7.8 | 1.3 |
|  | 19 | 335.7 | 200 | 67.14 | 1.98 | 8.2 | 1.3 |
|  | 26 | 297.8 | 200 | 59.56 | 1.99 | 8.6 | 1.2 |
| LGIN | 33 | 225.7 | 200 | 45.14 | 1.95 | 8.6 | 1.4 |
|  | 18 | 445.6 | 100 | 44.56 | 1.99 | 8.0 | 1.9 |
|  | 43 | 261.1 | 100 | 26.11 | 1.95 | 8.9 | 1.6 |
|  | 57 | 362.3 | 300 | 108.69 | 1.99 | 8.0 | 1.1 |
|  | 106 | 476 | 300 | 142.80 | 1.99 | 8.4 | 1.3 |
| HGIN | 118 | 252.5 | 200 | 50.50 | 1.96 | 8.4 | 1.3 |
|  | S 2 | 414.5 | 300 | 124.35 | 1.99 | 8.8 | 1.5 |
|  | 3 | 280.9 | 200 | 56.18 | 1.98 | 7.5 | 1.3 |
|  | 100 | 225 | 200 | 45.00 | 1.95 | 7.8 | 1.5 |
|  | 52 | 316.8 | 100 | 31.68 | 1.95 | 8.1 | 1.4 |
| CC | 10 | 1013.8 | 200 | 202.76 | 2.01 | 8.0 | 1.3 |
