# Supplementary material for: Comprehensive Analysis of Differentially Expressed Long Noncoding RNA-mRNA in the Adenoma-Carcinoma Sequence of DNA Mismatch Repair Proficient Colon Cancer
Source: J Oncol. 2021 Jun 1;2021:9977695. doi: 10.1155/2021/9977695 (PMC8208869; doi:10.1155/2021/9977695)
Supplement: Supplementary Materials — Additional file 1. Quality control results of sample RNA. Additional file 2. Quality control results of microarrays. [file 9977695.f1.zip › 9977695.f1/Additional file 2.docx]

**Additional file 2. Quality control results of microarrays**

| Microarrys | Samples | CV value (%) | Detection ratio (%) |
| --- | --- | --- | --- |
| NC-1 | 7 | 5.48794 | 61.13 |
| NC-2 | 8 | 5.67089 | 63.32 |
| NC-3 | 15 | 7.79072 | 60.49 |
| NC-4 | 16 | 5.58525 | 65.5 |
| NC-5 | 42 | 9.83843 | 61.1 |
| LGIN-1 | 19 | 5.03092 | 62.26 |
| LGIN-2 | 26 | 6.09747 | 62.43 |
| LGIN-3 | 33 | 5.42111 | 64.1 |
| LGIN-4 | 67 | 4.60446 | 58.24 |
| LGIN-5 | 135 | 10.4951 | 62.57 |
| HGIN-1 | 18 | 5.5907 | 59.38 |
| HGIN-2 | 43 | 6.03394 | 58.01 |
| HGIN-3 | 57 | 4.51835 | 60.53 |
| HGIN-4 | 106 | 4.92871 | 59.48 |
| HGIN-5 | 118 | 7.09983 | 59.31 |
| CC-1 | S 2 | 5.78829 | 59.8 |
| CC-2 | 100 | 4.97553 | 64.08 |
| CC-3 | 52 | 11.0553 | 61.62 |
| CC-4 | 10 | 10.6636 | 60.65 |
